# Affect in response to stressors and coping strategies: an ecological momentary assessment study of borderline personality disorder

**DOI:** 10.1186/s40479-017-0059-3

**Published:** 2017-05-21

**Authors:** Sadia R. Chaudhury, Hanga Galfalvy, Emily Biggs, Tse-Hwei Choo, J. John Mann, Barbara Stanley

**Affiliations:** New York State Psychiatric Institute/Columbia University, 1051 Riverside Drive, Unit 42, New York, NY 10032 USA

**Keywords:** Borderline personality disorder, Affect, Coping, Ecological momentary assessment

## Abstract

**Background:**

Affect instability is a core symptom of borderline personality disorder (BPD). Ecological momentary assessment allows for an understanding of real-time changes in affect in response to various daily stressors. The purpose of this study was to explore changes in affect in response to specific stressors and coping strategies in subjects with BPD utilizing ecological momentary assessment (EMA) methodology.

**Methods:**

Subjects (*n* = 50) with BPD were asked to complete real-time assessments about stressors experienced, affect felt, and coping strategies employed six times per day for a 1-week period. Mixed effect regression models were used to measure the effect of stressors and coping strategies on affect change.

**Results:**

While most stressors led to experiencing more negative affect, only being in a disagreement was independently associated with increased negative affect. Among coping strategies, only doing something good for oneself independently reduced negative affect, controlling for all other coping strategies used.

**Conclusions:**

These findings provide valuable insights into affective instability in BPD and can help inform treatment with individuals with the disorder.

## Background

Borderline personality disorder (BPD) is characterized by a pervasive pattern of instability in interpersonal relationships, self-image and emotion regulation that begin by early adulthood [1]. Difficulty in affect regulation is often identified as a core deficit in BPD [2–6]. Individuals with BPD display a wide arrange of negative emotions, from rage to sadness to shame. They often experience rapid mood transitions from one intense emotion to another, thereby experiencing numerous negative affects throughout the course of a given day [7]. Furthermore, once in an intense emotional state, individuals with BPD often find it difficult to recover [8]. Thus, evidence-based interventions for the treatment of BPD place a strong emphasis on acquiring effective coping strategies to counter and manage rapid emotional changes [8].

Much of what we know about affective instability and emotion dysregulation in BPD is based on ratings of recollections and self-report trait questionnaires. Such retrospective ratings are considered to be unreliable and sensitive to reporter bias [9, 10]. Recall bias among individuals with BPD appears to be negatively skewed; patients with BPD tend to overestimate the intensity of negative affect and underestimate the intensity of positive affect during retrospective mood ratings, while healthy volunteers have the opposite recall bias [11]. Ecological momentary assessment (EMA) provides a solution to this problem by allowing for real-time in vivo data collection of thoughts, feelings and behaviors [12]. EMA data collection is characterized by four key features: 1) data are only collected on very recent state to minimize recall bias; 2) repetitive measurements are taken to quantify how a given phenomenon changes over time; 3) the phenomena is measured at predefined intervals or situations; and 4) measurement takes place in the individual’s natural environment [9]. The use of EMA methodology has helped to develop a better understanding of BPD symptoms, including affective instability [11, 13–18]; dissociation [19]; interpersonal difficulties [20–22]; and suicidality [23–25].

Several EMA studies have shown that individuals with BPD exhibit greater variability and intensity of affect compared to both psychological healthy individuals [15, 17, 26] and other clinical populations [13, 27]. Specifically, individuals with BPD reported both a higher frequency and intensity of emotions than healthy controls during a 24-h psychophysiological ambulatory monitoring task, a form of EMA, where they were prompted every 10 to 20 min while awake to answer how they felt at the moment and the intensity of the feeling [26]. Individuals with BPD reported more negative affect and less positive affect than their healthy control counterparts, and also reported greater intensity of negative affect, but not positive affect, during the study. Compared to healthy controls, those with BPD were more likely to rapidly fluctuate from a positive to a negative mood state [11]. The affective instability found in individuals with BPD also distinguished them from other psychiatric populations [13]. When examined together, these findings lend strong support to the unique emotional dysregulation and affective lability struggles faced by those with borderline personality disorder.

These studies, however, did not capture what environmental factors contributed to the affective instability reported in their samples. In a study that used event-contingent sampling and paper-and-pencil diaries to understand variability in mood in response to interpersonal interactions in a sample of patients with BPD, individuals with BPD reported greater variability in positive affect following social interactions that lasted at least 5 min compared to healthy controls [20]. However, they reported comparable levels of variability in negative affect. While details on the participants’ interpersonal behavior were captured, information about the quality of the interaction and the participants’ feelings about the interaction (e.g., feeling rejected) were not. Participants with BPD also reported greater emotional reactivity in response to daily stress compared to patients with psychosis and healthy volunteers [27]. However, participants were only able to report on the single most stressful event that occurred during each time period, leaving the possibility that affective variability and dysregulation caused by other stressors during that time period were overlooked. Therefore, further research is needed to better understand the interplay between the environment and affect instability in BPD.

The present study sought to evaluate the emotional reaction to specified stressors by regular sampling throughout the day. How subjects with BPD respond to specific stressors, with what emotions and how they manage their response and emotions may help guide therapeutic interventions.

## Method

### Participants

Fifty participants who met DSM-IV-TR criteria for Borderline Personality Disorder were enrolled in this study. All participants provided written consent after receiving a detailed description of study procedures. The study protocol was approved by the Institutional Review Board at the New York State Psychiatric Institute. Eligibility criteria for the study included a diagnosis of borderline personality disorder and a history of either suicidal behavior or non-suicidal self-injury (at least one episode in the past 6 months and another within the past 2 years). Participants with bipolar I disorder, psychotic disorder, mental retardation, or an acute condition that required priority treatment, such as anorexia nervosa or severe substance dependence, were excluded. No comorbid Axis II diagnoses were excluded. The mean age of participants was 30.6 years (SD = 11, range: 18 to 62). As indicated in Table 1, the sample was predominantly female, Caucasian, and had a current diagnosis of Major Depressive Disorder. Eighty percent of the sample had a history of at least one suicide attempt, with an average of 2.2 suicide attempts (SD = 2.0) among attempters.

**Table 1:** Demographic and clinical characteristics of participants

*Demographic Characteristics*	Mean	S.D.	Range
Age	30.6	11.0	18-62
	Percent (n)		
Female	86% (43/50)		
White	56% (28/50)		
Single (never married)	82% (41/50)		
College graduate or above	46% (23/50)		
Currently Employed	58% (29/50)		
Abuse History			
Physical Abuse	22% (11/50)		
Sexual Abuse	20% (10/50)		
Physical and Sexual Abuse	26% (13/50)		
*Clinical Characteristics*			
	Percent (n)		
Current Major Depressive Disorder	62% (31/50)		
Lifetime History of Major Depressive Disorder	76% (38/50)		
History of Non-suicidal Self-Injurious Behavior	80.4% (37/46)		
History of Suicide Attempt	80% (40/50)		
	Mean	S.D.	Range
Number of Suicide Attempts	2.2	2.0	0-7

### Procedure

#### Baseline assessments

All participants completed a battery of assessments upon study entry with highly-trained Masters level psychologists who participated in regular reliability and consensus diagnostic conferences. Axis I and II psychopathology was diagnosed using the Structured Clinical Interviews for DSM-IV [28, 29]. Inter-rater reliability was high among assessors (ICC = 0.864) across Axis I and II disorders. The Beck Depression Inventory [30], a self-report measure of depressive symptoms was given to measure severity of depression at time of assessment.

#### Ecological momentary assessment

Participants were provided with a personal digital assistant (PDA) and completed a brief orientation session with a member of the research team. Participants practiced using the PDA with the assistant present until they were able to use the device on their own. They were instructed to carry the PDA with them at all times during a 1-week period and answer a series of questions when prompted. In order to best capture each participants’ day-to-day experiences, participants were asked to provide a 12-h period during which they expected to be awake and engaged in routine daily activities. During orientation, the PDA was customized to prompt each participant during their selected 12-h window. These prompts occurred on a random interval basis during a 12-h period. The daily 12-h window was divided into six two-hour blocks and one moment was randomly selected from each of these blocks for a total of six prompts over the course of each 12-h time period. Prompts were given randomly in order to avoid having participants anticipate when they would be asked to respond and to also ensure that a participant’s fixed schedule (e.g., work commitments) did not interfere with data collection on a regular basis.

At each prompt, participants were asked how strongly they experienced a series of affects since the last prompt using a 5-point Likert scale. The list of affects was derived from the Positive and Negative Affect Scales (PANAS) [31] and composite scores for positive and negative affect were calculated. In addition, participants were asked how overwhelmed they were by their feelings and to what degree their feelings felt out of control, using the same 5-point Likert scale. Participants were also asked whether any of the following stressors took place since the last prompt: 1) disagreement; 2) rejection; 3) compliment; 4) interpersonal disappointment; 5) neglect; 6) loss; 7) good news; 8) bad news; and 9) painful reminder from the past. Participants were able to indicate whether none, one, or several of these stressors took place during the assessment period. Finally, participants were asked if they used any of the following common coping strategies to manage the negative thoughts, feelings, or experiences they had since the last prompt: 1) keeping busy; 2) socializing; 2) positive thinking; 3) doing something good for self; 4) calming self; 5) finding perspective; and 6) sitting with feelings until they pass. The EMA prompts are detailed in Table 2. Similar to the questions about stressors, participants were able to indicate whether none, one, or several of the coping skills were employed. Participants also indicated whether they believed the coping strategies utilized were effective.

**Table 2:** Prompts used in EMA Survey

Category	EMA Question	Prompt	Item
Stressor	Since the last prompt, have you…	had a disagreement with someone?	Disagreement
		been rejected by someone?	Rejection
		been complimented or praised by someone?	Compliment
		been disappointed by someone?	Interpersonal disappointment
		felt neglected by someone?	Neglect
		experienced a loss of some sort?	Loss
		received good news?	Good news
		received bad news?	Bad news
		been reminded of something painful from the past?	Painful reminder
Coping Strategy	To what degree have you used the following strategies to manage any of the negative thoughts, feelings, or experiences you’ve had since the last prompt?	Kept myself busy	Keeping busy
		Socialized with others	Socializing
		Focused on positive thoughts	Positive thinking
		Did something good for myself	Doing something good for self
		Calmed myself down	Calming self
		Tried to find perspective	Finding perspective
		Sat with feelings until they passed	Sitting with feelings

### Statistical analysis

We analyzed the psychometric properties of the negative and positive affect scales from the EMA data following the recommendations of Shrout and Lane [32]. Our analysis is informed by the fact that the prompts occurred randomly, making observation time essentially nested within the subject, and also we have unequal number of observations per subject. Analysis of the internal consistency of the two affect scales over time was performed using random effect models to separate the proportion of variability explained by between subject variation, item by subject interaction, and time nested within subject. Due to the large number of items in each scale, for item-wise analyses only summaries are presented.

For our main analyses, we assessed the effect of stressors and coping strategies on the change in positive and negative affect using mixed effect regression models. Change in positive affect at a given time *t* was measured by the difference between the positive affect score at time *t* to the positive affect score at the previous time period, *(t-1)*, as long as both observations occurred on the same day. All analyses were performed using proc glimmix in the SAS™ software version 9.3 (Copyright@2002–2011, SAS Institute Inc., Cary, NC, USA), and the functions lme [33] and lmer in the statistical language R, version 2.12.1 [34]. Mixed effect regression model was fit with positive affect change as outcome, the time-varying covariate (s) as predictor, subject-specific random intercepts, and correlations between observations within the same subject modeled using an AR1 structure. An identical method was employed for negative affect. Time-varying predictors were stressors during the respective time interval; frequencies of coping skills employed during the respective time period, and self-rated effectiveness of coping skills. In secondary or sensitivity analyses, stressors were recoded in the form of change scores, namely, a new stressor in any epoch compared to the previous epoch was coded as “+1”, no change in stressor as “0”, and a stressor disappearing was coded as “−1”. A second set of sensitivity analyses adjusted for the effect of the length of the time lag between prompts as a fixed effect, and then using continuous time correlation structure for the residuals. For the emotion dysregulation outcomes (feeling overwhelmed, feeling out of control), the measure was on a Likert scale and was treated as an ordinal variable. Mixed effect proportional odds logistic regression was fit with subject-specific random intercepts for these two outcomes. The predictors were event indicators (yes/no) and frequencies of coping skills applied. Age was examined as a covariate and was not found to be significant. Significance levels were not adjusted for multiple testing.

## Results

There were 1448 EMA records across subjects during the 7-day assessment period. On average, there were 29 records per subject, approximately 4 per day. The time interval between prompts answered within the same day had a median value of 2.1 h, with Inter Quartile Range of 1.3–3.9 h.

The average correlation between items in the Negative Affect Scale was 0.34 (range: 0.17–0.67). The average correlation of items with the total negative affect score was 0.56 (range: 0.45–0.68). The estimate of between-person reliability of the negative affect scale items, averaged over time in the EMA context, was 0.9995, while the reliability of within-subject change in negative affect in time was 0.8474. Item-wise, the proportion of variance explained by the differences between subjects as opposed to within subject change ranged from 31% (for “Upset” and “Irritable”) to 62% (for “Scared” and “Afraid”). For the Positive Affect Scale, the, average pair wise correlation, calculated from mixed effect models, between the 10 items was 0.39 (range: 0.24–0.64); the mean correlation with the scale total was 0.60 (range: 0.57–0.65). The estimate of between-person reliability of the positive affect scale items, averaged over time in the EMA context, was 0.9991, while the reliability of within-subject change in positive affect in time was 0.8733. The inter-correlation of the two subscales, adjusted for intra-subject correlations, to be *r* = −0.21, (*t* = −9.14, *df* = 1327, *p* < 0.0001).

### Stressors as predictors of change in affect

We calculated change scores for negative and positive affect for observations within the same day, yielding a total of 979 measures for each. At each time point, participants reported an average of 2 stressors, with a range from 0 to 8, out of 9 possible stressors. Table 3 indicates the frequency of stressors reported, with “being reminded of something painful from the past” as the most commonly reported stressor.

**Table 3:** Stressors as a predictor of change in negative affect as measured by the difference in consecutive scores

Stressor	Frequency	Joint model			Individual models		
		B	t (*df* = 920)	*p*	B	t (*df* = 928)	*p*
Disagreement	20%	3.4336	5.92	<.0001	3.9110	7.82	<.0001
Rejection	16%	−0.7215	−1.04	0.2975	1.9642	3.75	0.0002
Compliment	22%	−0.9166	−1.89	0.0596	−0.7706	−1.64	0.1005
Interpersonal disappointment	29%	0.9671	1.63	0.1042	2.2006	5.28	<.0001
Neglect	30%	0.2081	0.40	0.6906	1.2837	3.14	0.0018
Loss	15%	−0.5124	−0.90	0.3697	0.9549	1.87	0.0616
Good news	17%	−0.1258	−0.23	0.8213	−0.6859	−1.28	0.2025
Bad news	16%	0.7375	1.25	0.2113	2.1786	4.00	<.0001
Painful reminder	45%	0.5763	1.41	0.1591	1.1752	3.16	0.0016

In the mixed effect regression model with multiple predictors, having a disagreement (*B* = 3.43; *t* = 5.92; *df* = 920; *p* < 0.0001), was found to be associated with increased negative affect, controlling for all other stressors. Further, receiving a compliment was associated on a trend level with decreased negative affect (*B* = −0.92; *t* = −1.89; *df* = 920; *p* = .0596). Single-predictor models for six of the seven negative stressor categories, disagreement, rejection, interpersonal disappointment, neglect, bad news, and a painful reminder from the past, were associated with significant increase in negative affect, while neither of the two positive stressors, receiving a compliment and receiving good news, was significantly associated with a change in negative affect (Table 3).

With regard to increasing positive affect, one of the two positive stressors, receiving good news, was found to be significant in both the multiple predictor (Good News: *B* = 1.95; *t* = 3.43, *df* = 920, *p* = 0.0006) and individual predictor models (*B* = 2.09; *t* = 3.92; *df* = 928; *p* < 0.0001;). No negative stressors were found to lead to significant decrease in positive affect in the individual or multiple predictor models.

Sensitivity analyses using a coding that separated new stressors from no change supported all the significant results above, additionally, some of the stressors gained significance; specifically, new incidents of interpersonal disappointment (*B* = 1.63; *t* = 3.53; df = 920; *p* = 0.0004), receiving bad news (*B* = 2.23; *t* = 4.63; df = 920; *p* < 0.0001), being reminded of something painful from the past (*B* = 1.71; *t* = 4.21; *df* = 920; *p* < 0.0001) were found to be associated with increased negative affect, and receiving a new compliment increased positive affect (*B* = 1.77; *t* = 4.02, *df* = 920, *p* < 0.0001 after controlling for all other events. A second set of sensitivity analyses tested the effect of the length of the time interval between consecutive prompts on the change in affect. First, we adjusted our joint model for testing the effect of life events on (negative) affect change by the time lag. The effect of time lag was not significant (*b* = −1.40, *SE* = 0.85, *z* = −1.65, *p* = 0.10); and neither of the life events’ effects changed substantially. Second, we let the intra-subject correlation in the mixed effect model vary based on the time lag; again, the results stayed substantially the same, preserving significance where detected in the primary analysis.

### Emotion dysregulation following a stressor

Participants were asked how overwhelmed they felt by their emotions, on a Likert scale from 1 to 5. In the mixed effect proportional odds logistic regression model with multiple predictors, four stressors were found to independently be associated with increased feelings of being overwhelmed: disagreement (OR = 2.70, *t* = 6.39, df = 1302, *p* < 0.0001); neglect (OR = 1.75, *t* = 3.81, df = 1302, *p* = .0001); bad news (OR = 1.92, *t* = 4.02, df = 1302, *p* < .0001); and painful reminder (OR = 2.08, *t* = 5.53, df = 1302, *p* < .0001). The two positive stressors were associated with decreased feelings of being overwhelmed in this model. Single-predictor models for each of the nine stressor categories indicated that all seven of the negative stressors were associated with an increase in feeling overwhelmed, while each of the positive stressors was associated with a decrease in feeling overwhelmed (Table 4).

**Table 4:** Stressors as a predictor of emotion dysregulation (mixed effect proportional odds logistic regression analysis)

Stressor	Joint model			Individual models		
	Odds ratio	*t* (*df* = 1302)	*p*	Odds ratio	*t* (*df* = 1310)	*p*
a. Stressors as a predictor of feeling overwhelmed						
Disagreement	2.70	6.39	<0.0001	4.76	11.12	<0.0001
Rejection	1.30	1.36	.1734	4.17	9.06	<0.0001
Compliment	0.74	−2.21	0.0276	0.68	−2.88	0.0041
Interpersonal disappointment	1.14	0.83	0.4095	3.33	9.66	<0.0001
Neglect	1.75	3.81	0.0001	4.98	9.10	<0.0001
Loss	1.28	0.13	0.1943	3.13	6.75	<0.0001
Good news	0.72	−2.19	0.0285	0.62	−3.35	0.0008
Bad news	1.92	4.02	<0.0001	3.57	8.59	<0.0001
Painful reminder	2.33	5.53	<0.0001	3.13	9.22	<0.0001
b. Stressors as a predictor of loss of emotional control						
Disagreement	2.65	6.10	<0.0001	4.76	10.79	<0.0001
Rejection	1.32	1.39	0.1660	4.17	8.84	<0.0001
Compliment	0.84	−1.19	0.2340	0.77	−1.87	0.0624
Interpersonal disappointment	1.43	2.11	0.0348	3.57	9.80	<0.0001
Neglect	1.64	3.16	0.0016	3.03	8.48	<0.0001
Loss	1.02	0.08	0.9342	2.63	5.61	<0.0001
Good news	0.93	−0.49	0.6214	0.78	−1.65	0.0993
Bad news	1.45	2.18	0.0298	2.78	6.78	<0.0001
Painful reminder	1.79	4.21	<0.0001	2.78	7.78	<0.0001

Participants were also asked to what extent they felt a loss of control over their emotions. In the mixed effect proportional odds logistic regression model with multiple predictors, five stressors were found to be independent risk factors for feeling a loss of emotional control: disagreement (OR = 2.63, *t* = 6.10, df = 1302, *p* < 0.0001); interpersonal disappointment (OR = 1.43, *t* = 2.11, *df* = 1302, *p* = 0.0348); neglect (OR = 3.06, *t* = 3.16, *df* = 1302, *p* = .0016); bad news (OR = 1.44, *t* = 2.18, *df* = 1302, *p* = 0.0298); and painful reminder (OR = 1.79, *t* = 4.21, *df* = 1302, *p* < .0001). Again, separate single-predictor models for each of the nine stressor categories indicated that all seven of the negative stressors were associated with feeling a loss of emotional control (Table 5), while each of the positive stressors was associated with a decrease in feeling this way.

**Table 5:** Coping strategies as a predictor of increase in positive affect as measured by the difference of consecutive scores

Coping strategy	Joint model			Individual models		
	B	*t* (*df* = 892)	*p*	B	*t* (*df* = 915)	*p*
Keeping busy	0.1511	0.98	0.3281	0.4154	3.21	0.0014
Socializing	0.2035	1.20	0.2286	0.5220	3.95	<.0001
Positive thinking	0.5510	2.25	0.0249	0.4822	3.07	0.0022
Doing something good for self	0.4402	1.97	0.0496	0.5634	3.61	0.0003
Calming self	−0.1799	−0.64	0.5251	0.01013	0.06	0.9520
Finding perspective	−0.6790	−2.52	0.0120	−0.07531	−0.48	0.6325
Sitting with feelings until they pass	0.05330	0.26	0.7919	−0.06201	−0.41	0.6823

### Coping strategies as predictors of decreased negative affect

Participants used on average 3.8 coping strategies per interval (S.D. = 2.4, range: 0 to 7). “Kept myself busy” was the most frequently used coping strategy (79%), while “calmed myself down” was the least commonly used (49%). The number of coping strategies applied was not associated with the length of time since the previous prompt (*b* = −0.33, *t* = −1.61, *df* = 914, *p* = 0.1078). Mixed effect regression models were used to test the effectiveness of each coping strategy on decreasing negative affect. In the multivariate model controlling for all of the other coping strategies, doing something good for oneself (*B* = −0.58, *t* = −2.54, df = 908, *p* = 0.0114) was the only significant independent predictors of decreased negative affect. In the single predictor models, two of the seven strategies were effective in decreasing negative affect: positive thinking (*B* = −0.34; *t* = −2.15; *df* = 915; *p* = 0.0321); and doing something good for self (*B* = −0.47; *t* = −2.97; df = 915; *p* = 0.0031). Sensitivity analyses showed that, when new coping strategies were separated out, positive thinking (*B* = −0.69, *t* = 0.13, *df* = 892, *p* = 0.0024) also had independent protective effect, while calming self (*B* = 0.80, *t* = 2.96, *df* = 892, *p* = 0.0032) and finding perspective (*B* = 0.89, *t* = 3.64, *df* = 892, *p* = 0.0003) both significantly increased negative affect.

### Coping strategies as predictors of increased positive affect

Mixed effect regression models were also used to test the effectiveness of each coping strategy on increasing positive affect. Several coping skills independently increased positive affect in the multiple predictor model, including, positive thinking, doing something good for oneself, and finding perspective (see Table 5). Four of the seven strategies were effective in increasing positive affect in the single predictor models: keeping busy, socializing, positive thinking, and doing something good for oneself.

## Discussion

While EMA methodology has been used previously to better understand the symptomatology of BPD, to our knowledge, this is the first study to utilize EMA methodology to explore in real-time both stressors that contribute to changes in affect and the coping strategies employed to manage the emotional response to these stressors in a sample of individuals with BPD. Participants faced a substantial number of daily stressors, the most common of which was a painful reminder of something from the past, which may be partially attributable to the substantial lifetime history of physical and sexual abuse in this sample. Other frequent stressors included feeling neglected and facing an interpersonal disappointment. The frequency of daily stressors suggests that individuals with BPD, with or without concurrent depression, face substantial pain and distress in their everyday lives.

We found increases in negative affect in response to most of the negative stressors; however, only disagreement was independently associated with increased negative affect, after controlling for all other stressors, although six of the seven negative stressor categories were associated with increased negative affect in single predictor models. Stressors associated with feeling overwhelmed were having a disagreement, feeling neglected, receiving bad news, and being reminded of something painful from the past. The aforementioned stressors all remained independent risk factors for feeling out of control, along with being disappointed by someone. Because the stressors occurred during the same time period as feeling overwhelmed or out of control, it cannot be determined whether the feeling or the stressor came first. Receiving good news was found to significantly predict increased positive affect in both individual and joint predictor models, serving as a reliability check for the survey responses. The sheer number of stressors associated with increased negative affect, decreased positive affect and emotion dysregulation speaks again to how stressful daily life can be for an individual with BPD. Several of the stressors associated with negative affect and emotion dysregulation are interpersonal in nature. This is not surprising given that interpersonal sensitivity is considered to trigger both the impulsive behaviors and the emotion dysregulation that are at the core of BPD [6, 35–38]. Consistent with our findings, social rejection and negative evaluation have been previously found to contribute to affective instability in individuals with BPD [37, 38].

Individuals with BPD employed a range of coping skills to manage their emotions. The most commonly employed strategies were keeping busy, finding perspective, and positive thinking. Positive thinking and doing something good for self both independently improved positive affect. Doing something good for self also independently reduced negative affect. These findings gives credence to the emotion regulation strategies encouraged by Dialectical Behavior Therapy and other similar therapies, which place an emphasis on self-soothing and self-care in response to managing difficult emotions [8]. The coping strategies of calming self, finding perspective, and sitting with feelings while they passed, while frequently used, were not helpful in this sample,. These strategies, particularly sitting with feelings until they pass, are similar to the mindfulness practice that is often prescribed for the treatment of BPD. However, it must be noted that these were individuals who were not in treatment and were not taught the skills in therapy; proper mindfulness training may lead to a more positive outcome with the use of this coping strategy, as indicated in other studies. Thus, our findings suggest that clinicians should be cautious when recommending this skill to their patients with BPD without adequate training. While it is not apparent why the coping strategies were more successful at increasing positive affect than decreasing negative fact, it is encouraging to find that these participants with untreated BPD were able to reduce their distress and improve their mood when faced with the numerous stressors they experienced on a daily basis.

This study is limited in several ways. The use of a 12-h window for data collection allows for the possibility that certain stressors, such as those occurring late at night, may not have been captured accurately. Our design of random prompts approximately every two hours does not allow for the detection of those affective changes that occur and disappear on a substantially shorter interval. The compliance rate for the overall sample during the weeklong assessment period was 69% (1,448 completed records from 2,100 prompts issued). While it is not known why participants answered the prompts at certain times and not others, it is possible that participants did not answer either when in crisis or when in a better mood, leading to the possibility of skewed results. Further, this study is limited to a BPD-only sample with no comparison control group. Thus, findings cannot be generalized to other clinical or psychiatrically healthy populations. Without exploring changes in affect in different clinical samples including psychiatrically healthy individuals, it is difficult to determine whether the response to stressors and coping strategies is unique to patients with BPD. This is particularly salient given that affective instability is not unique to BPD [39–42].

## Conclusions

This study provides a preliminary understanding of how stressors contribute to changes in affect in individuals with BPD, as well as how strategies used by these individuals help them cope with affective changes.  Further research is required to determine whether the changes in affect captured by EMA in this study in response to stressors or the coping strategies employed are unique to individuals with BPD, both in terms of frequency of affect change and intensity of affect experienced. In addition, future studies should continue to utilize real-time data to better understand the experiences of individuals with BPD in their actual environments, and potentially use this knowledge to tailor individualized treatments targeting their particular stressors and response to coping strategies.

## Abbreviations

BPD: Borderline personality disorder
EMA: Ecological momentary assessment
PDA: Personal digitial assistant
